# Particulate cartilage and platelet-rich plasma treatment for knee chondral defects in sheep

**DOI:** 10.1007/s00167-022-07295-7

**Published:** 2023-01-04

**Authors:** Lourdes Alcaide-Ruggiero, Verónica Molina-Hernández, Juan Morgaz, J. Andrés Fernández-Sarmiento, María M. Granados, Rocío Navarrete-Calvo, José Pérez, Setefilla Quirós-Carmona, José M. Carrillo, Ramón Cugat, Juan M. Domínguez

**Affiliations:** 1 Departamento de Medicina y Cirugía Animal. Facultad de Veterinaria, Universidad de Córdoba, Córdoba, Spain; 2 Fundación García Cugat para Investigación Biomédica, Barcelona, Spain; 3 Departamento de Anatomía y Anatomía Patológica Comparadas y Toxicología. UIC Zoonosis y Enfermedades Emergentes ENZOEM, Universidad de Córdoba, Córdoba, Spain; 4 Departamento de Medicina y Cirugía Animal, Universidad CEU Cardenal Herrera, Valencia, Spain; 5 Instituto Cugat y Mutualidad de Futbolistas Españoles, Delegación Catalana, Barcelona, Spain

**Keywords:** Articular cartilage, Chondrogenesis, Particulated cartilage, Platelet-rich plasma, Sheep

## Abstract

**Purpose:**

Articular cartilage is vulnerable to multiple types of damage and it has limited reparative and regenerative capacities due to its absence of vascularity. Although a large number of therapeutic strategies exist to treat chondral defects, they have some limitations, such as fibrocartilage formation. Therefore, the goal of the present study was to evaluate the chondrogenic regenerative properties of an autologous-made matrix of particulated cartilage and platelet-rich plasma (PACI + PRP) implantation for the treatment of full-thickness chondral defects in sheep.

**Methods:**

A full-thickness 8 mm diameter cartilage defect was created in the weight-bearing area of the medial femoral condyle in both knees of 16 sheep. The right knees of all animals were treated with particulated autograft cartilage implantation and platelet-rich plasma, while the left knees were injected with Ringer’s lactate solution or hyaluronic acid. The sheep were killed 9 or 18 months after surgery. Macroscopic evaluations were performed using three different scoring systems, and histopathological evaluations were performed using a modified scoring system based on different scoring systems.

**Results:**

The PACI + PRP groups showed statistically significant differences in the percentage of defect repair and chondrocytes in the newly formed cartilage tissue at 18 months compared to 9 months.

**Conclusions:**

The results suggest that macroscopic appearance, histological structure and chondrocyte repair were improved when using PACI + PRP treatment for chondral defects, producing an outcome similar to the surrounding healthy cartilage. PACI + PRP is a totally autologous, easy, and unexpensive treatment that can be performed in one-step procedure and is useful as a therapeutic option for knee chondral defects.

**Supplementary Information:**

The online version contains supplementary material available at 10.1007/s00167-022-07295-7.

## Introduction

Articular cartilage is a highly specialised tissue that provides a lubricious, low-friction gliding surface for joints [47]. Articular cartilage is vulnerable to trauma, overloading, ageing, and inflammation. In fact, these chondral injuries are some of the most common injuries of the musculoskeletal system and may lead to progressive damage and joint disorders such as osteoarthritis (OA) [43]. Cartilage has limited reparative and regenerative capacities due to its lack of vascularity [25]. In addition, mature chondrocytes lose their ability to migrate, proliferate, and synthesise their surrounding matrix [47]. The present therapeutic strategies applied to treat chondral lesions have some limitations, such as fibrocartilage formation without lasting improvements, prolonged recovery time, insufficient integration with healthy cartilage, the need to employ two-stage procedures, an inability to restore large defects, expensive treatments, or unpredictable results in athletes [10, 24, 37, 41, 44, 57]. Platelet-rich plasma (PRP) is a therapeutic option that is being increasingly used in musculoskeletal medicine. Its therapeutic potential is based on the supraphysiological supply of growth factors and cytokines, which promote the repair of tissues with low healing potential [12, 52, 54]. In vitro studies have reported that growth factors can stimulate chondrogenic regeneration, improving cartilage matrix protein biosynthesis and enhancing chondrocyte proliferation and metabolism [1]. In addition, PRP has been used in vivo to repair chondral defects in combination with other techniques, improving the quality of the repaired tissue [14, 38, 54, 58]. One of these techniques is particulated autograft cartilage implantation (PACI), which has been shown to lead to a better quality of repair of chondral lesions in animal and human studies [4, 8, 13, 14, 17]. However, the studies conducted to date have some relevant limitations, such as small sample size, the absence of comparative groups, short follow-up, and/or the absence of a meticulous study of the quality of the repaired tissue.

Therefore, the goal of the present study was to evaluate the chondrogenic regenerative properties of an autologous-based matrix composed of healthy hyaline cartilage chips with a clot of PRP and an intra-articular infiltration of PRP (PACI + PRP) for full-thickness defects in the weight-bearing area of the medial femoral condyle in sheep, and meticulous macroscopical and histopathological studies were carried out. It was hypothesised that this technique can restore chondral lesions in normal articular cartilage in sheep.

## Materials and methods

### Ethical statement

The present study was approved by the Bioethical Committee on Animal Research of the Regional Government of Andalusia (Junta de Andalucía 12/06/2016/109—reference SSA/SIS/MD/jv) and was conducted in accordance with the protection regulations for animals utilised for scientific purposes (Directive 2010/63/UE; Decision 2020/569/UE and RD 1386/2018).

### Animals and surgical procedure

Sixteen skeletally mature Merino sheep (*n* = 16), each weighing between 50 and 60 kg, were used for this study. A veterinary examination guaranteed that the animals were healthy and showed no musculoskeletal clinical signs before the surgical procedure, described as follows:

Both hind limbs of the anesthetised sheep were prepared for knee surgery. After a medial parapatellar approach, a 3–4 cm mini-arthrotomy was performed. The knees were flexed, and an 8 mm diameter punch was used to create a full-thickness cartilage defect in the weight-bearing area of the medial femoral condyle, without opening the subchondral bone. The right knees were treated with PACI + PRP, and the cartilage sample obtained was particulated in small 1–2 mm^3^ fragments and mixed with the activated PRP to obtain a clot used as a scaffold for the cartilage chips. A PACI + PRP matrix was placed to fill the cartilage defect. The adhesion of the clot was confirmed by performing flexion–extension of the knee. Then, the arthrotomy was closed, and 2 mL of activated PRP was injected intra-articularly. Moreover, the same surgical procedure for chondral defects was performed in the left knees, and the knees were randomly divided into two groups. Half of the left knees received an intra-articular injection of 2 ml of Ringer’s lactate solution (RLS, *n* = 8) as a control, and the other half were treated with 2 ml of hyaluronic acid (Synvisc One, Hylan G-F 20) (HA, *n* = 8). Finally, antibiotic (amoxicillin–clavulanic acid, 10 mg/kg IM) and analgesic (buprenorphine 0.02 mg/kg/8 h IM) treatments were given for 3 and 5 days after surgery, respectively.

Sheep were randomly divided into two study groups: the RLS/PACI + PRP (RLS) group, which included eight sheep treated with RLS in the left knee and PACI + PRP in the right knee (*n* = 8), and the HA/PACI + PRP (HA) group, which included eight sheep treated with HA in the left knee and PACI + PRP in the right knee (*n* = 8). Kill times were established at 9 or 18 months after surgery. Four randomly chosen sheep from each study group were killed at each time point.

### Platelet-rich plasma

PRP total treatment was prepared using the PRGF-Endoret system (Biotechnology Institute, Vitoria, Spain). Blood was collected in four extraction tubes from the jugular vein of each animal and was centrifuged for 8 min at 630 × *g* according to a published method [4]. Then, the centrifugated plasma volume was divided by 50%, so the upper layer and the deeper layer just over the buffy coat were labelled as fraction 1 and fraction 2, respectively. Fraction 2 was obtained by avoiding the aspiration of white and blood cells. The platelets were activated by adding 50 µL of calcium chloride 10% per 1 mL of plasma just prior to the use of both fractions. The whole treatment was studied, including PACI + PRP application and intra-articular PRP injection. For the PACI + PRP treatment, activated fraction 1 and fraction 2 were combined with the particulated cartilage at a 50/50 ratio and left for 30 min to obtain a semisolid scaffold. Additionally, a PRP intra-articular injection of 2 mL of activated fraction 2 was used.

The blood for PRP total treatment preparation was collected just prior to surgery and was processed and provided intraoperatively. The time delay between blood collection and the application of both fractions was less than 1.5 h. No postoperative PRP injection was given.

### Macroscopic evaluation

After killing at 9 or 18 months, digital high-resolution photographs were taken of the medial femoral condyle articular surface.

Macroscopic evaluation of cartilage repair was carried out according to three evaluation systems (Tables S1, S2, and S3). The validated International Cartilage Regeneration and Joint Preservation Society (ICRS) [50] scoring system analyses the degree of defect repair, the integration of the border zone, and the macroscopic appearance (Table S1). The evaluation described by Jung et al. [26] uses a semiquantitative score, which notes the filling of the defect, surface, integration, and colour (Table S2). The Goebel et al. [22] scoring system analyses the colour of the repair tissue, presence of blood vessels, surface, filling of the defect, and degeneration of adjacent articular cartilage (Table S3). In the three evaluation systems, the highest score is achieved for the best possible result. Scoring was performed by three different researchers for all three evaluation systems.

### Histopathological study

For the histopathological analysis, medial femoral condyles were harvested and fixed in 10% neutral buffered formalin for 24 h, decalcified, and then routinely processed and embedded in paraffin wax. Tissue sections (4 µm thick) were stained with haematoxylin and eosin (H&E). Histological images were taken using a photomicroscope (Olympus BX43), and photomicrographs were analysed with Image J software. To determine the quality of the repaired tissue, chondral defects were histologically evaluated and compared with the surrounding normal hyaline cartilage within the same sheep.

The regenerated tissue was scored using a modified scoring model based on different scoring systems [33, 34, 45, 51, 53] (Table 1). This scoring system included a total of 10 parameters. Six parameters were studied for a quantitative evaluation of chondrocytes and chondral repair: defect regeneration (%), cartilage thickness (µm), cell count (cell/mm^2^), lacunae area (µm^2^), cell area (µm^2^), and cell morphology (form factor = [π*area]/perimeter^2^). Four parameters were assessed for a semiquantitative evaluation of the cartilage structure: cartilage areas, tidemark formation, lateral integration of the defect, and cell distribution. The structure of the cartilage was scored on a scale of 0–14 (Table 1), with 14 points indicating a completely normal cartilage structure.

**Table 1:** Histological parameter analysed and score system used [33, 34, 45, 51, 53]

Histological parameter			Score system
Defect regeneration (%)			Pineda [33] and Wakitani [51]
Cartilage thickness (µm)			n/a
Cell count (cell/mm^2^)			n/a
Lacuna area (µm^2^)			n/a
Cell area (µm^2^)			n/a
Cell morphology (form factor = [π*area]/perimeter^2^)			ICRS II [34]
		Points	
Cartilage structure	Cartilage areas		ICRS II [34] and Mankin [53]
	Normal (superficial, middle, deep, and calcified zone)	4	
	Absence of one of the zones	3	
	Absence of two of the zones	2	
	Presence of only one zone	1	
	Total absence of cartilage	0	
	Tidemark formation		ICRS II [34] and Mankin [53]
	Yes, defined	2	
	Yes, blurred	1	
	No	0	
	Lateral integration of the defect		O´Driscoll [45]
	Complete integration of both sides	4	
	Full integration of one side	3	
	Partial integration of both sides	2	
	Partial integration of one side	1	
	No side integration	0	
	Cell distribution		Mankin [53]
	Normal	4	
	Diffuse hypercellularity	3	
	Diffuse hypercellularity and isogenic groups	2	
	Abundant isogenic groups	1	
	Hypocellularity	0	

n/a: not reported in previous studies

### Statistical analyses

A statistical analysis was performed using GraphPad Prism software 7.0 (Inc., San Diego, CA, USA). After performing the Kolmogorov–Smirnov normality test, the quantitative and semiquantitative variables were analysed using nonparametric tests. For the comparisons made in the same kill group, between treatments carried out in the same individual, the Wilcoxon test was performed. However, the Mann–Whitney U test was used to make comparisons between treatments carried out in different sheep. To compare the results of the same treatment group between the two kill periods, the Mann–Whitney U test was performed. The variables in the tables are expressed as means (minimum value – maximum value). The variables were considered statistically significant when the *p* value < 0.05*, *p* < 0.01**, or *p* < 0.001***.

Significant differences between groups as they pertained to the lacuna area were considered to be detectable, with a statistical power of 0.8 and, a significance level of 0.05. When considering a SD of 50 µm^2^ and a difference between groups of 100 µm^2^, 4 animals per group would be required.

## Results

All the animals reached the end of the study without incidents or adverse effects.

### Macroscopic appearance and scoring

After killing the animals, no gross changes consistent with inflammation or other pathological changes were observed in the knee joints.

At 9 and 18 months, a clear trend in the scores was observed in the macroscopic repair evaluation of the chondral defects treated with PACI + PRP, with the scores of the PACI + PRP knees being substantially higher according to the three evaluation systems used than those of their respective control knees treated with RLS or HA. Moreover, at 18 months after the administration of PACI + PRP treatment, the scores tended to be considerably higher than those obtained at 9 months for all of the studied groups, independent of the evaluation system used (Table 2).

**Table 2:** Score obtained from the macroscopic evaluation

Evaluation system	Time of killing	RLS	PACI + PRP (RLS)	HA	PACI + PRP (HA)	Healthy cartilage^Ѱ^
ICRS	9 months	2.6 (1.0–4.0)	7.6 (5.7–9.0)	4.2 (0.7–10.3)	6.6 (4.7–9.7)	12
	18 months	5.2 (4.0–6.3)	9.3 (6.7–11.7)	6.5 (5.3–8.3)	10.3 (8.0–11.7)	
GOEBEL	9 months	9.3 (5.3–12.0)	14.8 (13.3–16.3)	10.7 (4.7–17.0)	12.6 (9.7–16.0)	20
	18 months	11.2 (10.3–1.0)	16.6 (12.7–19.7)	14.8 (11.7–16.3)	17.8 (15.3–19.0)	
JUNG	9 months	1.8 (1.0–2.7)	4.0 (3.3–5.3)	2.1 (0.0–5.0)	3.0 (1.7–4.7)	6
	18 months	1.6 (1.0–2.3)	4.7 (2.7–6.0)	2.9 (1.7–4.0)	5.2 (3.7–6.0)	

RLS: left knee treated with Ringer’s lactate solution; PACI + PRP (RLS): right knee treated with particulated autograft cartilage implantation and platelet-rich plasma from the group treated with RLS in the left knee; HA: left knee treated with hyaluronic acid; PACI + PRP (HA): right knee treated with particulated autograft cartilage implantation and platelet-rich plasma from the group treated with HA in the left knee. Data are expressed as mean (minimum value–maximum value). ^Ѱ^Maximum score value

**Fig. 1:**
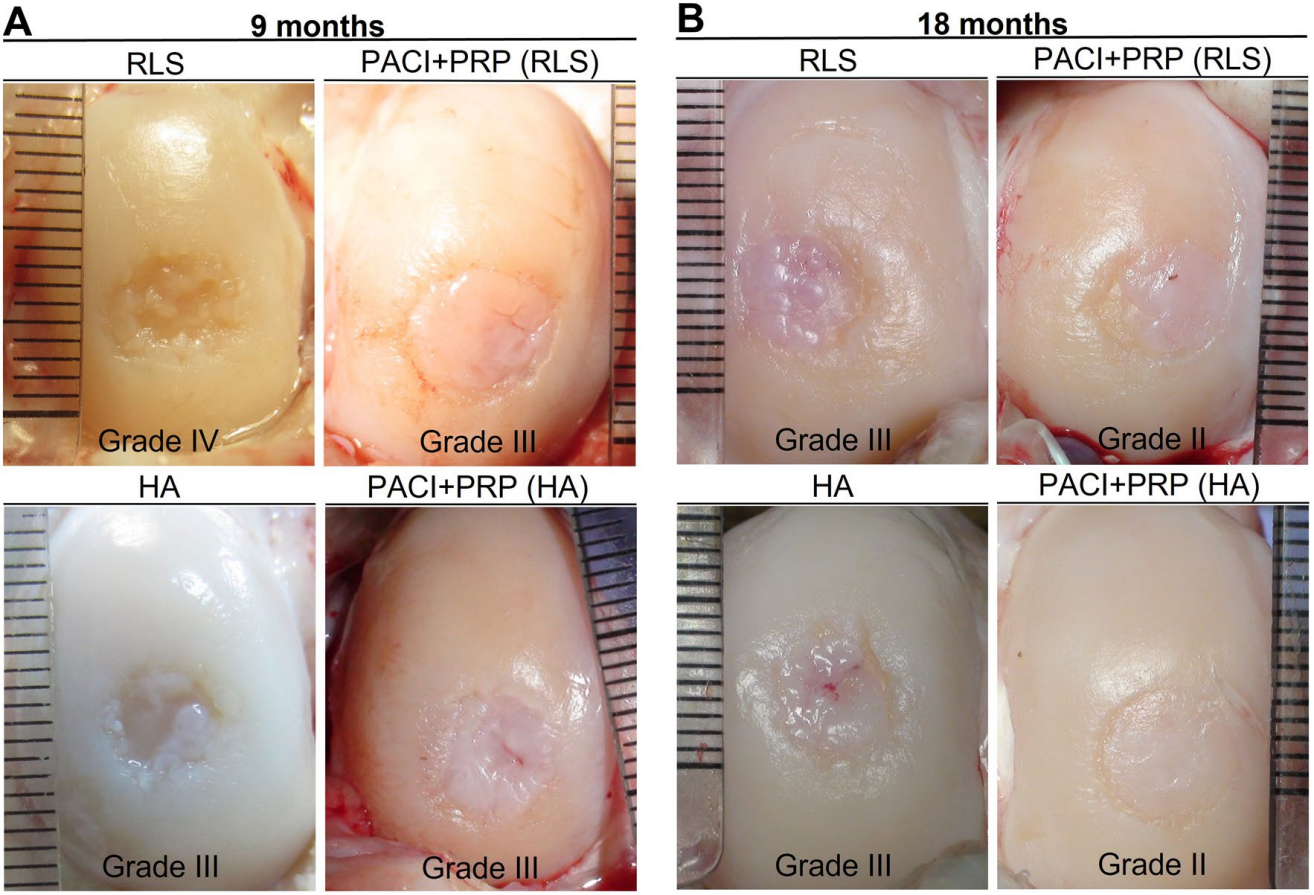
Macroscopic digital photographs of chondral defects after killing at 9 months (**A**) and 18 months (**B**) for RLS, PACI + PRP (RLS), HA, and PACI + PRP (HA) groups. Degree of repair according to the ICRS scoring system [50], with Grade II being nearly normal; Grade III being abnormal; Grade IV being severely abnormal. RLS: left knee treated with Ringer’s lactate solution; PACI + PRP (RLS): right knee treated with particulated autograft cartilage implantation and platelet-rich plasma from the group treated with RLS in the left knee; HA: left knee treated with hyaluronic acid; PACI + PRP (HA): right knee treated with particulated autograft cartilage implantation and platelet-rich plasma from the group treated with HA in the left knee

Furthermore, the ICRS scoring system allowed for the classification of the degree of chondral repair based on the score obtained (Table S1). The knees treated with RLS had the lowest degree of repair at 9 months (Fig. 1A). In addition, the PACI + PRP treated knees showed a nearly normal degree of repair at 18 months (Fig. 1B).

### Histopathological analysis

At 9 and 18 months, a percentage of the defect was regenerated, and the thickness of the cartilage regenerated with PACI + PRP tended to be closer to that of the normal hyaline cartilage among the three groups (Tables 3 and 4), showing the highest regeneration percentage at 18 months (90.4%). Likewise, the cell count, area of the lacuna, and cells of the regenerated cartilage did not differ between the treatments. The cell morphology values of the PACI + PRP groups tended to be closer to those of the surrounding normal hyaline cartilage, being equal at 18 months (0.93), and they were higher than those of the RLS and HA groups (Tables 3 and 4).

**Table 3:** Results of the histopathological analysis at 9 months (quantitative parameters)

9 months group								
	RLS	Hyaline cartilage RLS	PACI + PRP (RLS)	Hyaline cartilage PACI + PRP(RLS)	HA	Hyaline cartilage HA	PACI + PRP (HA)	Hyaline cartilage PACI + PRP(HA)
Defect regeneration (%)	56.1 (37.9–84.8)	100 (100–100)	64.4 (45.5–86.2)	100 (100–100)	44.9 (17.5–88.1)	100 (100–100)	68.5 (62.2–80.0)	100 (100–100)
Cartilage thickness (µm)	300.7 (175.5–368.1)	496.5 (452.5–525.0)	417.9 (365.6–507.2)	575.2 (499.8–713.2)	347.0 (241.7–446.9)	859.5 (385.2–1262.0)	333.5 (243.3–423.9)	461.0 (293.6–718.6)
Cell count (cell/mm^2^)	244.8 (223.8–262.9)	94.7 (69.9–107.2)	197.5 (163.5–229.1)	74.8 (68.4–85.1)	276.2 (177.9–381.1)	68.5 (54.6–87.7)	231.5 (214.6–256.1)	69.8 (51.3–91.2)
Lacunae area (µm)	102.6 (92.6–121.3)	208.3 (190.5–221.5)	103.2 (81.6–124.0)	193.7 (170.2–227.2)	77.5 (37.0–101.5)	154.5 (54.1–198.5)	97.9 (88.3–114.5)	220.9 (202.5–249.7)
Cell area (µm)	36.3 (32.9–39.7)	20.3 (16.7–25.4)	28.0 (24.7–31.9)	19.6 (18.3–20.2)	38.1 (24.7–45.5)	22.3 (17.1–31.3)	30.2 (26.6–33.5)	17.1 (15.6–20.0)
Cell morphology (form factor)	0.83 (0.78–0.86)	0.92 (0.91–0.93)	0.92 (0.89–0.93)	0.90 (0.88–0.93)	0.84 (0.82–0.85)	0.92 (0.90–0.95)	0.89 (0.86–0.91)	0.91 (0.90–0.92)

RLS: left knee treated with Ringer’s lactate solution; PACI + PRP (RLS): right knee treated with particulated autograft cartilage implantation and platelet-rich plasma from the group treated with RLS in the left knee; HA: left knee treated with hyaluronic acid; PACI + PRP (HA): right knee treated with particulated autograft cartilage implantation and platelet-rich plasma from the group treated with HA in the left knee. Data are expressed as mean (minimum value–maximum value)

**Table 4:** Results of the histopathological analysis at 18 months (quantitative parameters)

18 months group								
	RLS	Hyaline cartilage RLS	PACI + PRP (RLS)	Hyaline cartilage PACI + PRP(RLS)	HA	Hyaline cartilage HA	PACI + PRP (HA)	Hyaline cartilage PACI + PRP(HA)
Defect regeneration (%)	66.0 (50.1–82.0)	100 (100–100)	90.4 (78.4–99.3)	100 (100–100)	73.0 (27.9–90.2)	100 (100–100)	88.3 (74.9–96.7)	100 (100–100)
Cartilage thickness (µm)	287.2 (134.0–425.1)	639.6 (436.0– 808.9)	341.5 (271.5–461.8)	534.6 (408.4–701.7)	250.7 (53.5–471.3)	515.9 (229.4–690.2)	248.8 (118.6–339.1)	537.7 (181.2–938.6)
Cell count (cell/mm^2^)	218.5 (189.4–257.7)	69.3 (56.3– 88.7)	154.4 (141.2–170.5)	62.0 (56.6–67.7)	247.5 (130.4–377.8)	60.2 (45.3–72.3)	149.5 (129.6–165.0)	67.8 (57.6–82.8)
Lacunae area (µm)	82.4 (37.7–167.4)	121.2 (46.2- 200.7)	92.8 (42.8–146.2)	131.8 (51.4–215.7)	103.2 (40.7–142.7)	204 (189.3–224.1)	114.1 (39.8–162.6)	162.8 (49.8–223.6)
Cell area (µm)	27.0 (21.2–36.5)	20.7 (19.0–24.0)	24.3 (18.2–28.0)	19.9 (16.0–23.1)	30.2 (19.2–46.2)	20.7 (16.7–23.4)	20.0 (15.6–23.5)	21.1 (15.7–27.6)
Cell morphology (form factor)	0.89 (0.81–0.94)	0.93 (0.90–0.95)	0.93 (0.92–0.94)	0.92 (0.90–0.92)	0.89 (0.85–0.92)	0.92 (0.91–0.94)	0.93 (0.92–0.94)	0.93 (0.92–0.93)

RLS: left knee treated with Ringer’s lactate solution; PACI + PRP (RLS): right knee treated with particulated autograft cartilage implantation and platelet-rich plasma from the group treated with RLS in the left knee; HA: left knee treated with hyaluronic acid; PACI + PRP (HA): right knee treated with particulated autograft cartilage implantation and platelet-rich plasma from the group treated with HA in the left knee. Data are expressed as mean (minimum value–maximum value)

**Fig. 2:**
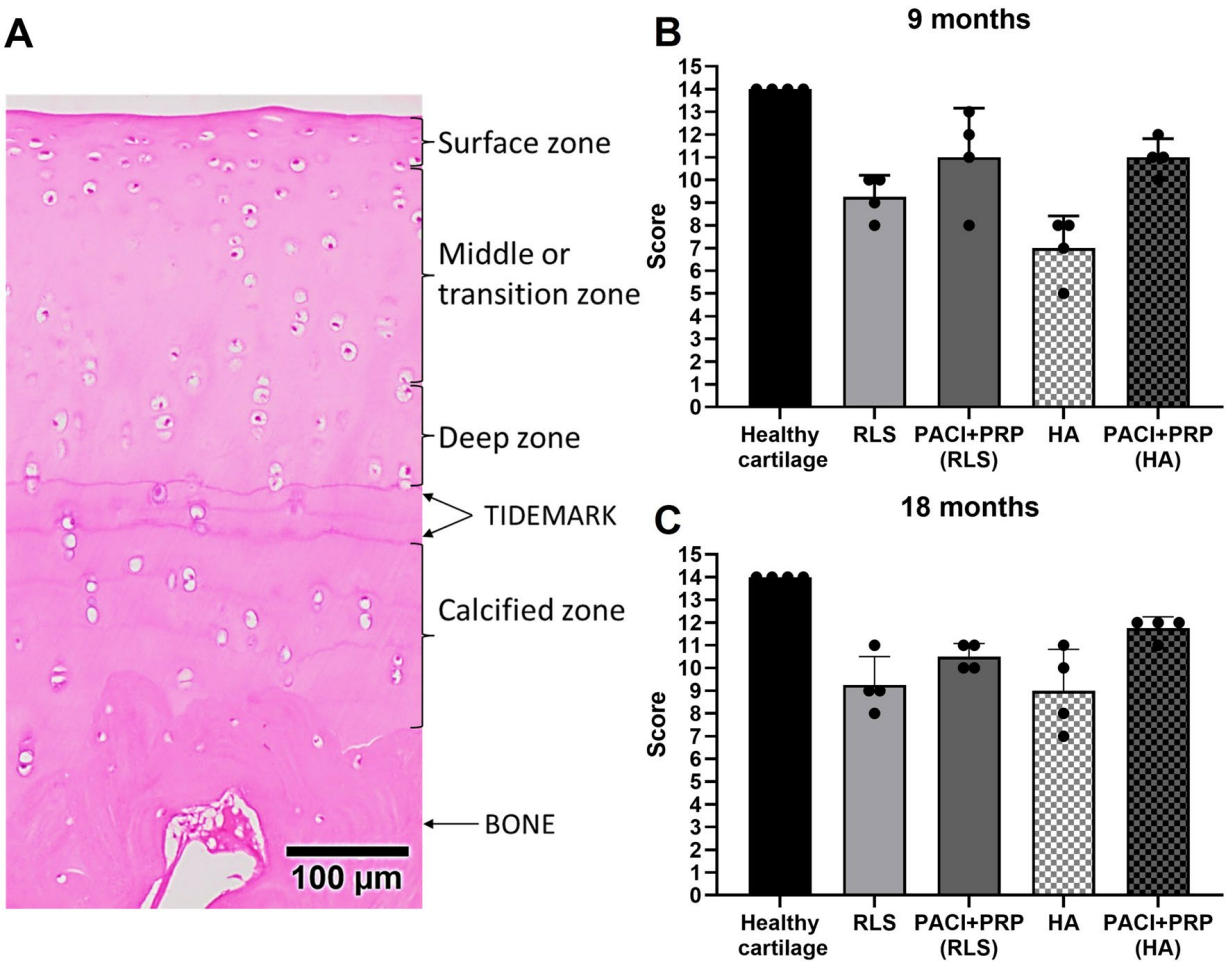
Analysis of the cartilage structure (semiquantitative parameters). **A** Histological image showing the different layers of healthy cartilage structure used to evaluate histopathological parameters (H&E stain). (**B** and **C**) Score obtained in each group evaluating the structure of the repaired cartilage at 9 and 18 months, respectively. RLS: left knee treated with Ringer’s lactate solution; PACI + PRP (RLS): right knee treated with particulated autograft cartilage implantation and platelet-rich plasma from the group treated with RLS in the left knee; HA: left knee treated with hyaluronic acid; PACI + PRP (HA): right knee treated with particulated autograft cartilage implantation and platelet-rich plasma from the group treated with HA in the left knee

**Fig. 3:**
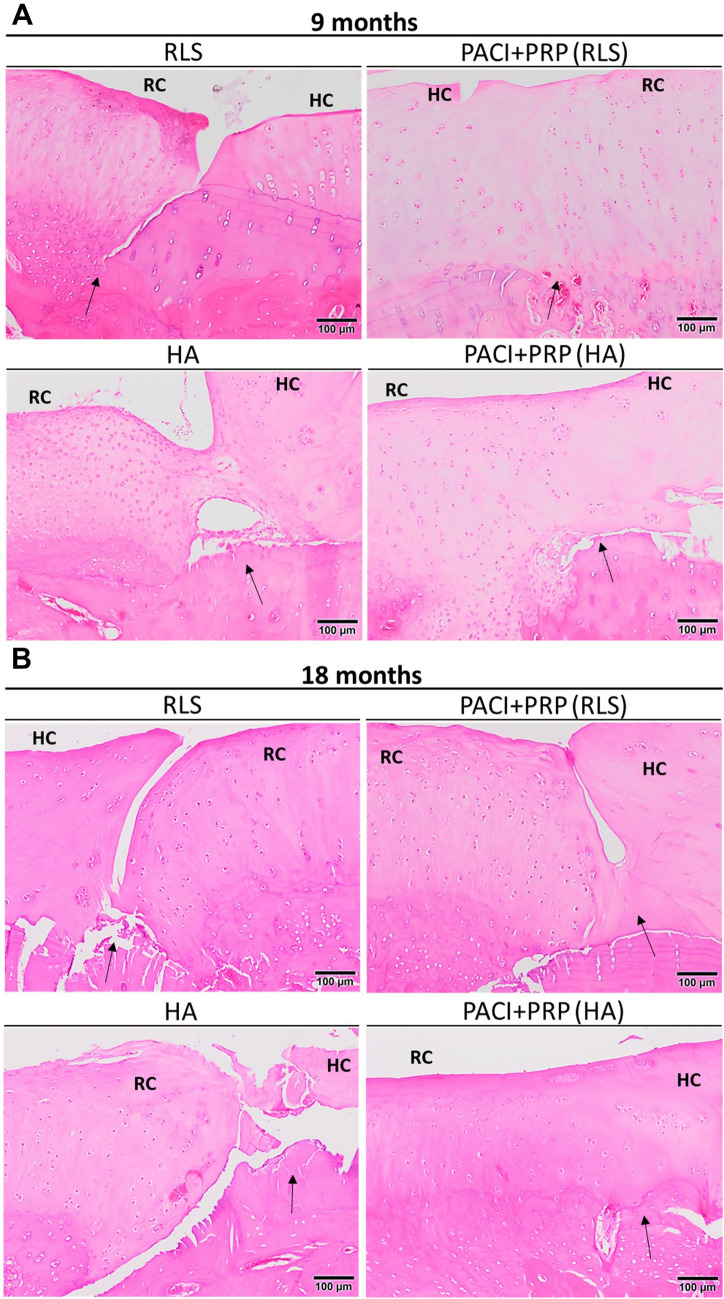
Histological images showing the structure of articular cartilage after administration of the different treatments at 9 and 18 months (H&E stain). The arrows indicate the boundary between healthy cartilage (HC) and repaired cartilage (RC), observing the degree of repair and lateral integration. **A** Histological images at 9 months showing complete lateral integration of the repaired cartilage with the surrounding healthy cartilage in PACI + PRP (RLS) and PACI + PRP (HA) groups, in contrast to the observation for the RLS and HA groups. **B** Histological images at 18 months showing better lateral integration of repaired cartilage with the surrounding healthy cartilage in PACI + PRP (RLS) group than in RLS group. Complete lateral integration of the repaired cartilage with healthy cartilage was observed in the PACI + PRP (HA) group compared with incomplete lateral integration of repaired cartilage in the RLS and HA groups. RLS: left knee treated with Ringer’s lactate solution; PACI + PRP (RLS): right knee treated with particulated autograft cartilage implantation and platelet-rich plasma from the group treated with RLS in the left knee; HA: left knee treated with hyaluronic acid; PACI + PRP (HA): right knee treated with particulated autograft cartilage implantation and platelet-rich plasma from the group treated with HA in the left knee

Regarding the evaluation of the cartilage structure, the PACI + PRP groups showed scores closer to those of the normal hyaline cartilage (Fig. 2). Despite not being statistically significant, the PACI + PRP-repaired cartilage was found to have better lateral integration with the surrounding cartilage, and the tidemark formation was more defined (Fig. 3). In terms of cellular organisation, no major differences were observed between the different groups.

### Chondrocytes and chondral repair: recovery at 9 and 18 months

**Fig. 4:**
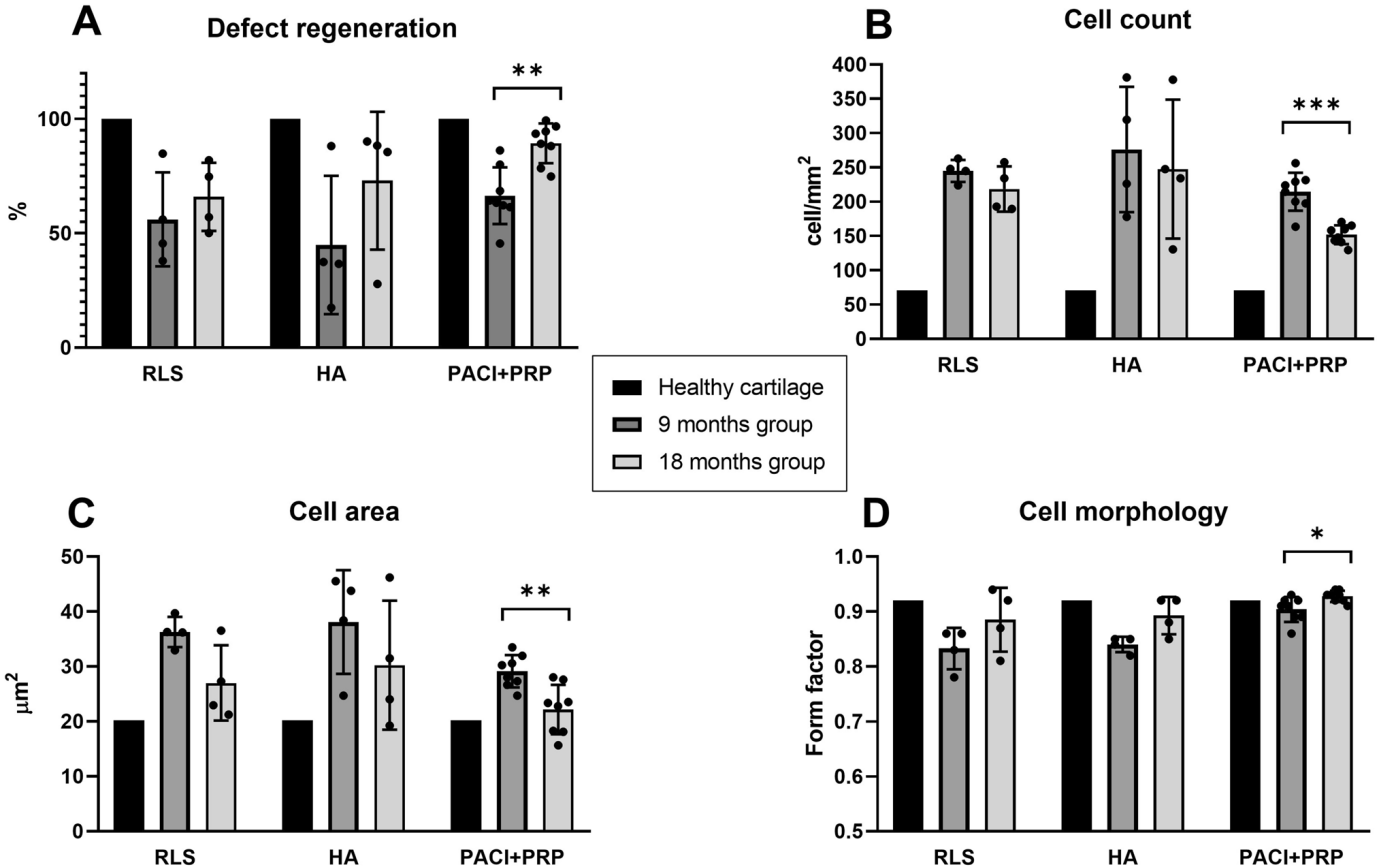
Comparison of the different treatments according to percentage of cartilage defect repair and chondrocyte recovery time studied (9 and 18 months). **A** Defect regeneration (%). **B** Cell count (cell/mm^2^). **C** Cell area (µm^2^). **D** Cell morphology (form factor). RLS: left knee treated with Ringer’s lactate solution; HA: left knee treated with hyaluronic acid; PACI + PRP: right knee treated with particulated autograft cartilage implantation and platelet-rich plasma. *p < 0.05; **p < 0.01; ***p < 0.001

The sheep treated with PACI + PRP showed statistically significant increases in the percentage of regenerated defect (25.6%; *p* = 0.002) (Fig. 4A) and cell morphology (0.02 form factor; *p* = 0.021) (Fig. 4D) in repaired cartilage tissue at 18 months compared to 9 months of recovery. Statistically significant decreases were observed in cell count (Fig. 4B) (64.7 cell/mm^2^; *p* = 0.001) and cell area (7.0 µm^2^; *p* = 0.005) (Fig. 4C) in cartilage regenerated with PACI + PRP treatment at 18 months compared to 9 months of recovery.

## Discussion

Considering the key role of chondrocytes in the maintenance and repair processes of hyaline cartilage, the most important finding of the present study was the improved quality observed in the cartilage cells in the PACI + PRP groups, which were very similar to those of healthy cartilage.

Despite improvements in technology and tissue engineering, an effective treatment for articular cartilage repair remains elusive [25, 39]. Techniques such as autologous chondrocyte implantation (ACI) or matrix-induced ACI (MACI) require two surgical interventions, in addition to cell cultures, and they have possible drawbacks, such as cell senescence and dedifferentiation [9, 18, 35, 37, 48]. The autologous matrix-induced chondrogenesis technique (AMIC) needs to be combined with other techniques [40]. Despite reporting substantial improvements, the obstacles surrounding cell-based techniques have led experts to study and use other therapeutic strategies. A possible alternative is the application of small cartilage chips that have been previously particulated or minced (PACI or MCI) [2, 31, 32, 36]. This technique has the advantages of being able to be applied in a single surgical intervention and not requiring cell cultures or scaffolds. A growing number of animal and human studies support the potential of better repaired cartilage quality using minced or particulated cartilage techniques [2, 8, 11, 21, 23, 32, 35, 56]. These promising results stem from the migration of chondrocytes into a biomaterial and the subsequent cartilaginous extracellular matrix (ECM) deposition by these cells [19].

In this study, a therapy for chondral lesions based on the combination of PACI and autologous growth factor treatment was presented. In this method, the autologous hyaline cartilage chips serve as a bioactive matrix. Cugat et al. [15] and Delman et al. [16] showed in studies with humans that PACI is a safe and efficient surgical procedure with promising results. Therefore, the goal of this study was to provide in vivo evidence on chondrogenic repair quality following treatment with PACI + PRP by carrying out a detailed study of the quality of repaired tissue in sheep over two prolonged periods of time, 9 and 18 months after surgery, and by comparing the results with those of RLS and HA treatments.

The degree of fragmentation in particulated cartilage is a critical parameter for the amount of ECM production [29], which is hypothetically due to chondrocyte activation upon the mechanical stimulation of the cut [7, 45]. Bonasia et al. [7] recommended fragmenting cartilage into cubes of about 2 mm^3^ or using minced cartilage. Farr et al. [20] carried out a study comparing particulated cartilage and minced cartilage, concluding that both techniques appear to be similar. In this study, the cartilage particles were fragmented into cubes that were between 1 and 2 mm^3^ in size. Another important consideration is the material used as a scaffold to support the particulated cartilage. No consensus exists on the nature of the most appropriate biomaterial to be used to promote chondrocyte migration [5]. Fibrin glue is the material most often used as a scaffold in chondral lesions treated with particulated cartilage [16, 21, 23, 28, 42, 46, 55]. However, the source and concentration of its components vary widely, affecting both its mechanical strength and adhesive properties [6]. When PRP was used as scaffold, it promoted cell migration but also served as bioactive scaffolds to promote chondrocyte viability, proliferation, and differentiation, acting as reservoirs of growth factors and cytokines [12, 27]. Furthermore, growth factors and cytokines released by PRP have been shown to play a key role in chondrogenesis during cartilage repair [4, 59]. A 100% autologous bioactive matrix was used in PACI + PRP treatment, which combined hyaline cartilage chips that can generate a chondrogenic environment once mixed with PRP clots, and allowing the construct to reach a semisolid state before implantation. The further intra-articular injection of PRP after scaffold implantation provides an additional source of growth factors to enhance the regenerative joint environment within the defect.

The quality of cartilage formed in a chondral defect treated with PACI + PRP in sheep was investigated in this study, and exhaustive macroscopic and microscopic analyses were carried out over a long period of time after surgery. In this study, an attempt was made to obtain more robust results. For this reason, different validated assessment systems were used. Furthermore, the assessment of some other parameters (cartilage thickness, cell count, lacuna area, and cell area) was considered important, so these were added after the literature review was performed for this study.

The macroscopic evaluation showed that the PACI + PRP groups had a better appearance than the RLS and HA groups, especially after 18 months of recovery. The cartilage obtained in the RLS group showed the worst macroscopic appearance. The histological analysis showed that the cartilage repair tissue structure in the PACI + PRP groups was of a higher quality than that in the RLS and HA groups at both 9 and 18 months of recovery. Furthermore, the chondrocytes showed a statistically significant more advanced repair process in the PACI + PRP groups after 18 months of recovery. It is recognised that chondrocytes play a unique role in the development, maintenance, and repair of ECM [3].

In this study, most of the observed results were not statistically significant despite the promising results of the cartilage being repaired to a nearly normal state after 18 months of treatment with PACI + PRP. This is probably due to the great variability that exists between sheep [30, 49], so a large dispersion of the data were found. However, in the present study, some of the limitations of previous studies were able to overcome. Control groups were included to compare the results of the treatments at different times, and a longer follow-up study was also carried out.

## Conclusion

The macroscopic and histological structure evaluations of cartilage repair showed that the newly formed tissue after the PACI + PRP treatment tended to be more similar to healthy articular cartilage than that after RLS and HA treatments at 9 and 18 months. The percentage of cartilage defect repair and chondrocytes resulted in a statistically significant and more advanced repair process in the PACI + PRP groups after 18 months of recovery.

## Supplementary Information

Below is the link to the electronic supplementary material.

Supplementary file1 (DOCX 16 KB)

## Data Availability

The data presented in this study are available on request from the corresponding author.

## Abbreviations

ACI: Autologous chondrocyte implantation
AMIC: Autologous-matrix-induced chondrogenesis
ECM: Extracellular matrix
HA: Hyaluronic acid
HC: Healthy cartilage
ICRS: International Cartilage Regeneration and Joint Preservation Society
MACI: Matrix-induced ACI
MCI: Minced cartilage implantation
OA: Osteoarthritis
PACI: Particulated autograft cartilage implantation
PJAC: Particulated juvenile articular allograft
PRP: Platelet-rich plasma
RC: Repair cartilage
RLS: Ringer’s lactate solution
