# Supplementary material for: Particulate cartilage and platelet-rich plasma treatment for knee chondral defects in sheep
Source: Knee Surg Sports Traumatol Arthrosc. 2023 Jan 4;31(7):2944–55. doi: 10.1007/s00167-022-07295-7 (PMC10275813; doi:10.1007/s00167-022-07295-7)
Supplement: Supplementary file 1 — Supplementary file1 (DOCX 16 KB) [file 167_2022_7295_MOESM1_ESM.docx]

**SUPLEMENTARY MATERIAL**

**Table S1, S2, and S3.**

**Table S1.** ICRS macroscopic evaluation of repair [50].

| **Cartilage repair assessment ICRS** | **Points** |
| --- | --- |
| Degree of defect repair |  |
| In level with surrounding cartilage | 4 |
| 75% repair of defect depth | 3 |
| 50% repair of defect depth | 2 |
| 25% repair of defect depth | 1 |
| 0% repair of defect depth | 0 |
| Integration to border zone |  |
| Complete integration with surrounding cartilage | 4 |
| Demarcating border <1 mm | 3 |
| 3/4th of graft integrated, 1/4th with a notable border >1mm width | 2 |
| 1/2 of graft integrated with surrounding cartilage, 1/2 with a notable border >1 mm | 1 |
| From no contact to 1/4th of graft integrated with surrounding cartilage | 0 |
| Macroscopic appearance |  |
| Intact smooth surface | 4 |
| Fibrillated surface | 3 |
| Small, scattered fissures or cracks | 2 |
| Several, small or few but large fissures | 1 |
| Total degeneration of grafted area | 0 |
| Overall repair assessment |  |
| Grade I: normal | 12 |
| Grade II: nearly normal | 11 – 8 |
| Grade III: abnormal | 7 – 4 |
| Grade IV: severely abnormal | 3 – 1 |

**Table S2.**  Adaptation of Jung´s semi-quantitative score [26].

| **Parameter** | **Points** |
| --- | --- |
| Defect filling |  |
| Empty | 0 |
| Half | 1 |
| Complete | 2 |
| Defect surface |  |
| Rough | 0 |
| Smooth | 1 |
| Defect integration |  |
| Bad | 0 |
| Good | 1 |
| Defect colour |  |
| Dark | 0 |
| Bright | 1 |
| Normal | 2 |
| Total score | 6 |

**Table S3.** Adaptation of Goebel macroscopic evaluation of repair [22].

| **Parameter** | **Points** |
| --- | --- |
| Color of the repair tissue |  |
| Hyaline or white | 4 |
| Predominantly white (>50%) | 3 |
| Predominantly translucent (>50%) | 2 |
| Translucent | 1 |
| No repair tissue | 0 |
| Presence of blood vessels in the repair tissue |  |
| No | 4 |
| Less than 25% of the repair tissue | 3 |
| 25-50% of the repair tissue | 2 |
| 50-75% of the repair tissue | 1 |
| More than 75% of the repair tissue | 0 |
| Surface of the repair tissue |  |
| Smooth, homogenous | 4 |
| Smooth, heterogeneus | 3 |
| Fibrillated | 2 |
| Incomplete new repair tissue | 1 |
| No repair tissue | 0 |
| Filling of the defect |  |
| In level with adjacent cartilage | 4 |
| >50% repair of the defect depth or hypertrophy | 3 |
| <50% repair of defect depth | 2 |
| 0% repair of defect depth | 1 |
| Subchondral bone damage | 0 |
| Degeneration of adjacent articular cartilage |  |
| Normal | 0 |
| Cracks and/or fibrillations in integrations zone | 1 |
| Diffuse osteoarthritic changes | 2 |
| Extension of the defect into the adjacent cartilage | 3 |
| Subchondral bone damage | 4 |
| Total score | 20 |
